# Loss of SeqA confers low-level fluoroquinolone resistance through transcriptional reprogramming and RpoS dependence in *E. coli*

**DOI:** 10.3934/microbiol.2025047

**Published:** 2025-12-24

**Authors:** Amir Faraz, Nuha Abeer Khan, Deepak Kumar Singh, Hamna Syed, Mohan C. Joshi

**Affiliations:** Multidisciplinary Centre for Advanced Research and Studies (MCARS), Jamia Millia Islamia, New Delhi, India-110025

**Keywords:** SeqA, *Escherichia coli*, Fluoroquinolones (FQs), antibiotic resistance, replication initiation, DNA double-strand breaks (DSB), SOS response, RpoS

## Abstract

SeqA is a key regulator of DNA replication initiation and chromosome cohesion in *Escherichia coli*. Loss of SeqA causes replication asynchrony, segregation defects, and growth delay, but its role in antibiotic susceptibility has remained unclear. Fluoroquinolones (FQs), which directly target bacterial DNA gyrase and topoisomerase IV to generate double-strand breaks (DSBs), provide a useful system to probe how chromosomal organization influences antibiotic response. In this study, we investigated whether SeqA loss alters sensitivity to FQs compared to antibiotics with non-DNA targets. MIC and MBC assays revealed that Δ*seqA* cells exhibit a specific low-level resistance to FQs, with ~1.5-fold higher inhibitory and bactericidal thresholds while retaining *wildtype* sensitivity to β-lactams and aminoglycosides. Using MuGam-GFP and RecA-GFP reporters, we showed that Δ*seqA* cells had fewer DSBs and mount an attenuated SOS response at *wildtype* MIC levels, enabling survival at otherwise lethal doses. Complementation restored *wildtype* sensitivity, confirming SeqA's direct involvement. Importantly, resistance was abolished in Δ*seqA-rpoS* double mutants and upon sub-MIC rifampicin treatment, demonstrating that RpoS-dependent transcriptional reprogramming underlies this phenotype. This suggested that Δ*seqA* strains acquire resistance through an RpoS-dependent regulatory effect that likely involves broad transcriptional reprogramming that underlies this phenotype. Together, these results showed that loss of SeqA alters chromosome organization in a way that lowers fluoroquinolone-induced DNA damage and enables RpoS-dependent low-level resistance.

## 1. Introduction

SeqA is a key DNA-binding protein in *Escherichia coli* that regulates replication initiation and chromosome organization. It preferentially binds hemimethylated GATC sites immediately after replication, sequestering *oriC* to prevent premature reinitiation [1],[2]. SeqA is conserved in *Enterobacteriaceae* but is absent in many other Gram-negative bacteria, where it cooperates with Dam methylase to support multifork replication during rapid growth in nutrient-rich media [3]. Studies have shown that under nutrient-poor conditions, where multifork replication is absent, loss of SeqA has little effect; however, in nutrient-rich conditions, ∆*seqA* cells display increased doubling time, pronounced segregation defects, and abnormal cell phenotypes, indicating that replication defects are exacerbated during rapid growth [4],[5]. Beyond replication control, SeqA forms higher-order nucleoprotein complexes that compact DNA, mediate transient sister chromatid cohesion, and facilitate proper chromosome segregation [6]. In addition, SeqA modulates local DNA supercoiling and influences transcriptional regulation, including the expression of topoisomerases such as *gyrA*, *gyrB*, and *topA*, thereby linking chromosome organization with stress responses and cell cycle progression [5],[7],[8]. Loss of SeqA leads to asynchronous replication initiation, chromosome segregation defects, and frequent replication fork collapse, resulting in elevated DNA damage [9],[10]. Collectively, SeqA functions as a multifaceted regulator that safeguards genomic integrity and promotes genome stability in *E. coli*. Although SeqA is non-essential, Δ*seqA* mutants become susceptible to DNA double-strand breaks in the absence of recombination repair [9], suggesting that these strains may also be sensitive to DNA-targeting antibiotics.

Fluoroquinolones are bactericidal antibiotics that induce DNA double-strand breaks by stabilizing cleavage complexes of DNA gyrase and topoisomerase IV, enzymes essential for DNA replication and transcription, leading to the accumulation of lethal DSBs [11]–[13]. At the minimal inhibitory concentration (MIC), the number of DSBs reaches a threshold that activates the SOS response, the primary pathway for DSB repair [14]. The SOS response is initiated when DNA double-strand ends are resected by the RecBCD complex, generating 3′ single-stranded DNA overhangs. These overhangs are coated by the recombinase RecA, forming presynaptic nucleoprotein filaments. This complex triggers autocleavage of the LexA repressor, which normally inhibits the SOS regulon. Once LexA is cleaved, a variety of SOS-regulated genes are activated, including those encoding error-prone DNA polymerase and cell division inhibitor (e.g., *sulA*), enabling the cell to respond to oxidative stress [15]. While activation of the SOS response is often monitored by increased expression of the *recA* gene or other SOS‑regulated genes such as the *sulA* promoter [16], research has shown that the MuGAM protein fused with GFP can be used to detect and count double‑strand breaks (DSBs) directly in cells [17]. In addition to the SOS response, cells also activate a general stress response regulated by *rpoS* (σ^S^), which controls transcription of genes important for survival under many stress conditions, including nutrient limitation, oxidative stress, osmotic imbalance, acid stress, and stationary-phase entry [18],[19]. Upon encountering DNA damage, RpoS can modulate the expression of a subset of genes, including antioxidant systems and DNA protection proteins, to complement the SOS-mediated repair response, thereby enhancing overall stress tolerance and promoting cell survival [20],[21].

Resistance to FQs in bacteria generally arises in a stepwise manner; initially bacteria acquire low-level quinolone resistance (LLQR) and subsequently evolve high-level quinolone resistance (HLQR) if selection pressure continues [22]–[24]. LLQR typically arises from single target-site mutations, plasmid-mediated quinolone resistance (PMQR) genes, or modest efflux activity and often remains within the clinically susceptible range. In contrast, HLQR results from the stepwise accumulation of multiple mechanisms, including combinations of target-site mutations, efflux pump overexpression, and multiple PMQR determinants, which elevate minimum inhibitory concentrations (MICs) above clinical breakpoints and lead to therapeutic failure [22],[25]. Therefore, LLQR represents an early adaptive state, involving regulation of DNA replication, genomic reorganization, and altered gene expression, conferring subtle tolerance phenotypes and accelerating the stepwise evolution toward high-level resistance. Since SeqA plays a central role in coordinating replication timing, maintaining fork stability, ensuring proper chromosome segregation, and preserving genomic integrity, its loss may influence early adaptive responses, including LLQR. Therefore, investigating the fate of LLQR in SeqA deficient strains is critical not only for understanding the earliest evolutionary steps of fluoroquinolone resistance but also for elucidating the contribution of replication and chromosome organization processes to antibiotic resistance.

In this study, we screened the MIC of Δ*seqA* strain across classes of antibiotics. Our findings reveal that Δ*seqA* strains exhibit specific low-level resistance to fluoroquinolones (LLQR), with an ~1.5-fold increase in MIC and MBC while remaining sensitive to β-lactams and aminoglycosides. At the *wildtype* FQ MIC, Δ*seqA* strains display reduced DSB formation and mount a lower SOS response, enabling cells to survive. This low-level resistance to FQs is reversed in Δ*seqA*-*rpoS* double mutants as well as upon treatment with sub-MIC rifampicin. These findings suggest that altered chromosome topology and gene-expression contribute to the low-level resistance phenotype.

## 2. Materials and methods

### 2.1. Strains and chemicals

The *E. coli* K-12 strain MG1655 (MJ 35) was used as the *wildtype* reference strain in this study. The isogenic deletion mutants Δ*seqA* (MJ 379) and Δ*seqA*-*rpoS* double mutant (MJ 1053) were constructed via P1 phage transduction using gene knockouts from the Keio collection, which contains single gene deletion mutants with kanamycin resistance cassettes. Transduced cells were selected on LB agar plates containing 50 µg/mL kanamycin. All strains were maintained on LB agar and stored in 25% glycerol stocks at −80 °C. For all experiments, strains were grown in M9 minimal medium supplemented with 0.2% (w/v) D-glucose (M9-glucose), prepared using M9 salts (5×) purchased from Sigma-Aldrich (USA). Overnight cultures were grown in M9-glucose and diluted 1:1000 into fresh medium for sub-culturing. All cultures were incubated at 37 °C with shaking at 200 rpm unless otherwise specified. Ciprofloxacin (≥ 98% purity), colistin sulfate, and other antibiotics were obtained from Sigma-Aldrich. Ciprofloxacin was prepared as a 10 mg/mL stock solution in sterile water and stored at −20 °C. Unless otherwise stated, all experiments were performed using three independent biological replicates, each initiated from cultures grown on separate days. Each biological replicate was measured in triplicate technical replicates. For RT-qPCR, each biological replicate was further analyzed using three technical replicates per reaction.

### 2.2. Determination of minimum inhibitory concentration (MIC)

MIC was determined with slight modifications to the CLSI guidelines (CLSI M7-A8) [26]. As noted, the Δ*seqA* mutant exhibits abnormal phenotypes and segregation defects in nutrient-rich media such as LB or Mueller–Hinton broth under standard conditions. Instead of the prescribed nutrient-rich Mueller–Hinton broth, we used M9 minimal medium supplemented with glucose (0.2%). The rationale for this modification was that, in rich medium, *ΔseqA* cells exhibit abnormal morphology, slow growth, and lower cell density (CFU), whereas no such defects are observed in minimal medium [4]. This difference is likely due to increased asynchronous origin firing and fork collisions in rich media compared to minimal media [5]. Therefore, minimal medium was used to avoid confounding effects of growth media. Six independent single colonies from freshly streaked LB plates of both strains were grown to saturation in M9-glucose at 37 °C for 16 hours. The overnight cultures were diluted 1:100 into fresh M9-glucose medium and incubated for 3 hours, typically reaching an OD₆₀₀ of 0.2. Cells were then dispensed into 96-well U-bottom microplates containing serial dilutions of antibiotics. Growth was monitored at 37 °C with continuous orbital shaking using a BioTek 800 TS microplate reader (600 nm), with OD₆₀₀ measurements recorded hourly for 15 hours. MIC values were defined as the lowest antibiotic concentration that inhibited visible growth after 15 hours of incubation. Each condition was performed in triplicate, and no-antibiotic controls were included for each strain to normalize growth kinetics.

### 2.3. Determination of minimum bactericidal concentration (MBC)

Following the 15-hour incubation with or without antibiotics, cultures were washed in 1× PBS, serially diluted and plated on M9-glucose agar plates. Plates were incubated at 37 °C for 16 hours, and colonies were counted to determine the lowest antibiotic concentration that caused 99.9% bacterial killing, defined as the MBC [27]. This experiment was performed five times independently, each with more than three technical replicates, and the data are presented as mean ± SD.

### 2.4. Fluorescence microscopy

Strains (MJ609, MJ1264, MJ1051, and MJ1052) were cultured in M9-glucose at 37 °C for 16 hours. Overnight cultures were diluted 1:100 into fresh M9-glucose medium and incubated for 3 hours, typically reaching an OD₆₀₀ of 0.2, after which different concentrations of ciprofloxacin (0, 1, 5, 10, 15, and 25 ng/mL) were added. Cells (200 µL) were harvested at 0, 2, and 4 hours after ciprofloxacin treatment by centrifugation at 5,000 rpm for 15 minutes at 4 °C. The cell pellets were washed with phosphate-buffered saline (PBS, pH 7.5) and fixed with 2.5% paraformaldehyde for 15 minutes at room temperature, followed by 45 minutes at 4 °C. Cells were then washed thrice with 1× PBS and resuspended in 20 µL of 1× PBS and incubated for 15 minutes at room temperature prior to imaging. For imaging, cells were mounted on 1.5% agarose pads prepared with M9 salts. Fluorescence microscopy was performed using a Zeiss Axio Observer 7.0 inverted microscope equipped with a 100× oil immersion objective (Plan-Apochromat, NA 1.40), appropriate filter sets for GFP, and a monochrome CCD camera. Representative images were captured using Zen Blue imaging software in. czi format, with defined ROIs exported as .*tiff* files. For quantitative analysis of MuGam-GFP foci per cell and RecA-GFP intensity per cell, images were exported in .tiff format and analyzed using ImageJ software (NIH) or Zen analysis tools [28]. A minimum of 800 cells per experiment were analyzed across time points and treatments.

### 2.5. Flow cytometry

Flow cytometry was performed to quantify DNA breaks and SOS response in *E. coli* strains expressing MuGam-GFP (MJ609 & MJ1264) and RecA-GFP (MJ1051 & MJ1052), respectively, at the population level. Strains were grown in M9-glucose medium and treated with different concentrations of ciprofloxacin (0, 1, 5, 10, 15, and 25 ng/mL). Cells were harvested at 0, 2, 4, 6, and 8 hours of exposure by centrifugation (5000 × g, 5 minutes), washed three times with phosphate-buffered saline (1× PBS, pH 7.5), and fixed with 2.5% paraformaldehyde for 15 minutes at room temperature followed by 45 minutes at 4 °C. Fixed cells were washed and resuspended in 1× PBS and analyzed using a CytoFLEX flow cytometer (Beckman Coulter) equipped with a 488 nm blue laser and standard GFP detection filters with identical instrument settings (laser power, detector voltage, and compensation) for all samples. Gating thresholds were established using the 0 h untreated (0 ng/mL ciprofloxacin) sample of each strain to define the GFP-negative population. Subsequent samples were analyzed using this gate, and the proportion of GFP-positive events was determined relative to each strain's own untreated control. This per-strain normalization strategy was adopted to account for minor differences in baseline GFP fluorescence between strains, thereby avoiding false-positive classification of basal signal as induced expression. The dotted vertical line shown in histogram plots represents the mean fluorescence intensity (MFI) of the untreated control sample, providing a consistent reference point across all treatments. A minimum of 100,000 events were recorded per sample in triplicate. Gating for GFP-positive populations was established using untreated (0-hour) control samples to define baseline fluorescence. Data were analyzed with FlowJo software.

### 2.6. β-Galactosidase assay for SOS response

To quantify SOS response activation, a strain with a chromosomally integrated *sulA*-*lacZ* reporter fusion in *wildtype* (MJ1130) and Δ*seqA* (MJ1131) strains were used. β-galactosidase activity was measured as described previously [29]. Strains were grown in M9-glucose medium and treated with ciprofloxacin at 0 ng/mL (control), 5 ng/mL, 15 ng/mL, and 25 ng/mL with or without rifampicin (1 ug/mL). After 2 hours of antibiotic exposure, 1 mL of culture was harvested by centrifugation (5000 × g, 5 minutes), washed once with Z-buffer (60 mM Na₂HPO₄, 40 mM NaH₂PO₄, 10 mM KCl, and 1 mM MgSO₄) and resuspended in 1 mL of the same buffer. Cells were permeabilized with 50 µL chloroform and 20 µL 0.1% SDS, followed by incubation at 28 °C for 5 minutes. The enzymatic reaction was initiated by adding 200 µL of 4 mg/mL o-nitrophenyl-β-D-galactopyranoside (ONPG) and stopped with 500 µL of 1 M Na₂CO₃ after color development. The absorbance of the yellow product (o-nitrophenol) was measured at 420 nm, and β-galactosidase activity was calculated in Miller Units using formula {Miller Units = 1000 × [(OD420 - 1.75 × OD550)]/(T × V× OD600)}. Untreated samples (0 ng/mL) served as negative controls to define a baseline expression. The assay was performed in triplicate, and results were reported as mean β-galactosidase activity ± standard deviation.

### 2.7. Quantitative real-time PCR (qRT-PCR)

Total RNA was extracted from *E. coli* cultures using the RNeasy Mini Kit (Qiagen) following the manufacturer's protocol, after 2-hour ciprofloxacin treatment (5, 15 and 25 ng/mL) or untreated controls. RNA was treated with DNase I (Thermo Fisher) to eliminate genomic DNA contamination. cDNA was synthesized from 1 µg RNA using the iScript cDNA Synthesis Kit (Bio-Rad). Quantitative PCR was performed using SYBR Green Master Mix (Bio-Rad) on a CFX96 Real-Time PCR Detection System (Bio-Rad). Gene-specific primers were used for *rpoS*, *ydiJ*, and *gcvR*, and the housekeeping gene *lacI* served as an internal control. Relative gene expression was calculated using the ΔΔCt method. All reactions were performed in triplicates from at least three independent biological replicates.

### 2.8. Statistical analysis

Statistical analyses were performed using GraphPad Prism. For comparisons between two groups, unpaired two-tailed Student's t-tests were used, and statistical significance was defined as p < 0.05. All values are presented as mean ± SD. Each experiment included three independent biological replicates, with three technical replicates per biological sample. For RT-qPCR, each biological replicate was analyzed with three technical replicates per reaction.

## 3. Results

### 3.1. Loss of SeqA confers low level fluoroquinolones resistance in *E. coli*

SeqA is a well-known protein involved in controlling replication initiation, mediating site-specific cohesion along the chromosome and stabilizing the replisome [6],[9],[30]. However, its role in *E. coli* under antibiotic stress remains untested. Fluoroquinolones (ciprofloxacin, norfloxacin, and levofloxacin) were selected because they directly target bacterial DNA by stabilizing DNA topoisomerase cleavage complexes, leading to replication-associated double-strand breaks. In contrast, β-lactams (imipenem) and aminoglycosides (spectinomycin), along with additional controls such as colistin and chloramphenicol, were included as comparator drugs that target processes unrelated to DNA replication such as cell wall biosynthesis, protein synthesis, or membrane integrity, enabling us to determine whether any observed phenotype was specific to DNA-targeting agents. To test this, we determined the Minimal Inhibitory Concentration (MIC) as well as the Minimal Bactericidal Concentration (MBC) of representative antibiotics in a strain lacking SeqA and compared them to the *wildtype* strain. For MIC and MBC determination, we used a slightly modified CLSI broth microdilution protocol (as discussed in material method section). To assess whether the observed differences in susceptibility were influenced by medium composition, we compared the growth of *wildtype* and Δ*seqA* strains in LB, Mueller–Hinton broth and M9–glucose. Δ*seqA* exhibited pronounced growth and CFU defects in LB and Mueller–Hinton media, whereas both strains grew comparably in M9–glucose (Figure S1). These findings are consistent with previous reports that *seqA* mutants experience replication stress and fork collapse under rapid growth conditions in nutrient-rich media [5],[31]. Therefore, M9–glucose medium was used for all susceptibility assays to maintain comparable replication dynamics and minimize confounding effects from growth-rate differences.

**Figure 1.**
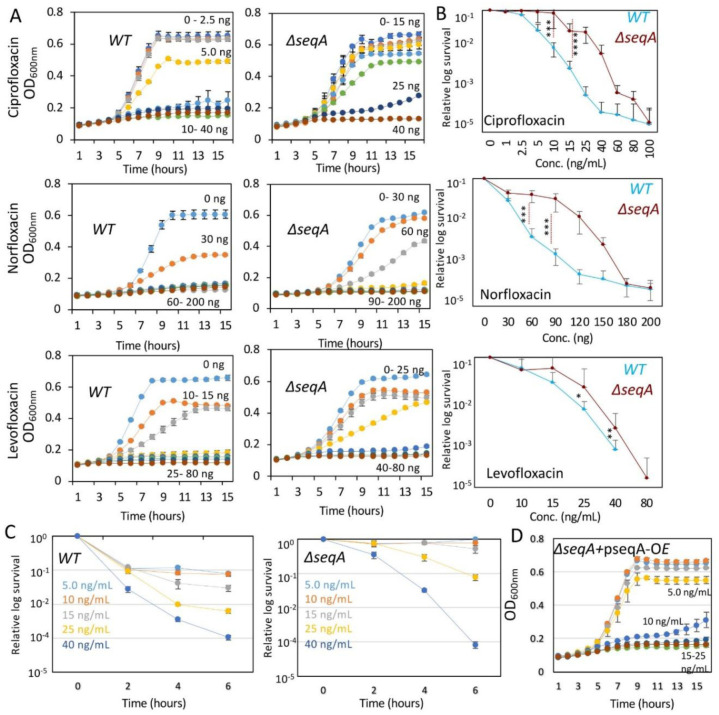
Loss of SeqA confers increased resistance to fluoroquinolones in *E. coli*. (A) Growth kinetics of *wildtype* (*WT*) and Δ*seqA* strains in M9-glucose minimal medium supplemented with increasing concentrations of ciprofloxacin (0–25 ng/mL), norfloxacin (0–200 ng/mL), or levofloxacin (0–180 ng/mL). Δ*seqA* strains maintain higher growth at inhibitory concentrations compared to *WT*. (B) Corresponding survival assays (CFU counts) showing dose-dependent killing in *WT* but enhanced survival of Δ*seqA* across all tested fluoroquinolones. (C) Time-kill kinetics of *WT* and Δ*seqA* strains exposed to ciprofloxacin at 5–40 ng/mL. Δ*seqA* mutants exhibit reduced killing over time, consistent with increased resistance. (D) Genetic complementation of the Δ*seqA* mutant with a SeqA-overexpression plasmid restores ciprofloxacin sensitivity, confirming that resistance arises specifically from loss of *seqA*. *, **, and *** represent the significance p-value of 0.01, 0.001, and 0.0001, respectively. Data points are average of at least 3 independent experiments. Data points represent the mean of three independent biological replicates; each measured in triplicate technical replicates. Error bars represent standard error. Statistical significance was determined by Student's t-test (***p < 0.001).

Our analysis revealed that MIC and MBC values for Δ*seqA* were largely comparable to those of the *wildtype*, with two notable exceptions: Fluoroquinolones (FQs) and chloramphenicol. The Δ*seqA* strain exhibited a low-level resistance phenotype (~1.5-fold increase in MIC relative to *wildtype*) against ciprofloxacin (25 ng/mL), norfloxacin (90 ng/mL), and levofloxacin (40 ng/mL). In contrast, loss of SeqA resulted in increased sensitivity to imipenem (200 µg/mL), comparable growth inhibition against spectinomycin (40 µg/mL), and colistin (1 µg/mL) (Figure 1A and Figure S2A). Interestingly, Δ*seqA* also showed increased sensitivity to chloramphenicol, with its MIC decreasing from 5 µg/mL in the *wildtype* to 2 µg/mL in the mutant strain (Figure S2A).

Consistently, doubling time analysis further highlighted fluoroquinolone-specific effects. Moreover, while both strains exhibited similar increases in doubling time when treated with non-DNA-targeting drugs, *wildtype* cells exposed to sub-MIC levels of ciprofloxacin displayed a dramatic increase in doubling time. In the presence of sub-MIC ciprofloxacin (10 ng/mL), the cell-division time increased to ~503 +/− 0.86 minutes in *wildtype* cells, whereas in Δ*seqA* it was ~162 +/− 0.46 minutes (Figure S2A). CFU analysis also corroborated these findings. Consistently, the ~1.5-fold MIC increase correlated with a two-fold MBC increase for fluoroquinolones in Δ*seqA* (from 40 to 80 ng/mL) (Figure 1B and Figure S1B and S2C). To assess whether the higher MIC reflected true resistance rather than transient tolerance, we performed time-dependent killing assays with ciprofloxacin [32]. *Wildtype* cells were rapidly killed within 2–4 hours at 25 ng/mL ciprofloxacin, whereas Δ*seqA* mutants exhibited markedly delayed killing kinetics at both 15 and 25 ng/mL (Figure 1C). Even at 40 ng/mL, where both strains were killed, Δ*seqA* retained higher viability at early time points. This survival under continuous drug exposure demonstrates that SeqA loss confers true resistance, not merely tolerance. To directly test whether the resistance phenotype was attributable to SeqA loss, we complemented the mutant strain with recombinant SeqA, overexpressing plasmid (pSeqA-OE). SeqA supplementation restored ciprofloxacin MIC in Δ*seqA* to *wildtype* levels (Figure 1D). Importantly, this resistance phenotype was growth-phase dependent. When cultures were treated with ciprofloxacin in the stationary phase, both strains showed comparable susceptibility, and no significant difference in survival was observed. This indicated that the Δ*seqA*-associated resistance is specific to actively replicating cells where replication-linked processes are engaged (Figure S3B).

**Figure 2.**
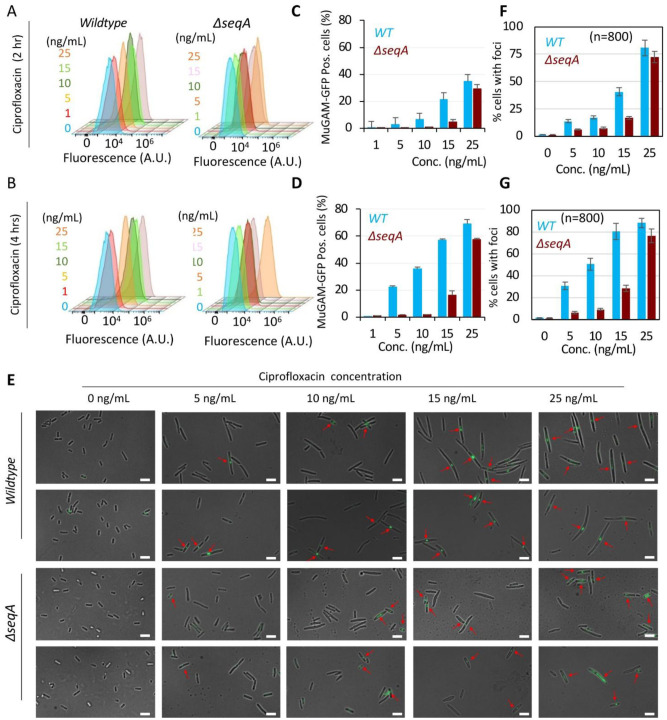
Deletion of *seqA* reduces ciprofloxacin-induced DNA double-strand breaks (DSBs) in *E. coli*. Flow cytometry histograms show the percentage of MuGam-GFP positive cells—indicative of DSBs—in *wildtype* (*WT*) and Δ*seqA* strains treated with 0, 1, 5, 10, 15, and 25 ng/mL ciprofloxacin for (A, C) 2 hours and (B, D) 4 hours. (E) Representative fluorescence microscopy images of MuGam-GFP foci (green) in *WT* and Δ*seqA* strains following treatment. (F–G) Quantification of cells with MuGam-GFP foci after 2-hour and 4-hour ciprofloxacin treatment, respectively. Data represent the mean ± SD of three independent biological replicates.

Together, these results indicate that the absence of SeqA reduces cellular susceptibility to fluoroquinolones, likely by altering chromosomal topology and replication dynamics in a manner that lowers the frequency of lethal gyrase–DNA cleavage events. Thus, the organized chromosomal architecture maintained by SeqA in *wildtype* cells, while essential for replication fidelity, increases vulnerability to fluoroquinolone-induced DNA damage. Finally, SeqA complementation restored MIC and MBC values supporting the conclusion that low-level resistance to FQs arises from replication stress during active growth.

### 3.2. SeqA mutant requires higher ciprofloxacin concentrations than wildtype to induce comparable levels of fluoroquinolone-induced DNA Double-Strand Breaks and SOS response

Since Δ*seqA* displayed a selective increase in fluoroquinolone MIC and MBC, we next asked whether this phenotype reflected altered DNA damage formation. Because fluoroquinolone lethality is tightly linked to the accumulation of drug-stabilized double-strand breaks (DSBs), quantifying DSBs was the logical next step to understand the mechanistic basis of the resistance phenotype. The mode of FQ-induced cell death involves random DNA double-strand breaks (DSBs) across the genome. This occurs because FQs trap bacterial topoisomerases (DNA gyrase and topoisomerase IV) in a ternary complex with DNA, thereby preventing the relegation of DNA ends [11],[22]. During normal growth, cells also experience DSBs primarily due to replication fork collapse or the generation of endogenous reactive oxygen species (ROS). Cells possess mechanisms to counteract these DSBs, either through homologous recombination (HR)-mediated repair or by reducing intracellular ROS levels via the expression of antioxidant proteins [33]. However, when the number of DSBs exceeds the repair capacity, cells trigger the SOS response which regulates the expression of more than 50 genes, including those involved in mutagenic repair, septum formation and other stress responses [15],[34]. To investigate the mechanism underlying the Δ*seqA* mutant–mediated low-level resistance to FQs, we examined the extent of DSB induction and the degree of SOS activation following FQ treatment compared to *wildtype* strains. For this purpose, we employed two reporter systems: MuGam-GFP and RecA-GFP.

MuGam is a bacterial homolog of the eukaryotic Ku70 protein, which binds tightly to DNA DSB ends. When fused to GFP, MuGam forms distinct foci at sites of DSBs in living cells, making MuGam-GFP a highly specific and direct reporter of DSB formation in bacteria [17]. To monitor DSBs, we integrated a temperature-inducible cassette, PR*gam*-*gfp* (attT7::FRT*cat*FRT λcI^ts^857 PR*gam*-*gfp*), a kind gift from Prof. Susan Rosenberg, into *wildtype* and Δ*seqA* strains. Upon shifting to 37 °C, MuGam-GFP is constitutively expressed and the formation of foci reflects DSB frequency, as MuGam binds to DNA ends and prevents their degradation. Although RecA expression alone does not directly indicate SOS induction, the processing of DSB ends by the RecBCD complex generates ssDNA, which becomes coated with RecA to form a presynaptic filament. This filament promotes the autocleavage of the LexA repressor, thereby activating the SOS response [33],[35]. Thus, RecA expression serves as an indirect readout of SOS induction via LexA cleavage. However, RecA-GFP fusions often compromise RecA activity. To overcome this, we used a merodiploid strain (a kind gift from Dr. Christian Lesterlin) carrying *wildtype recA* and *recA*-*GFP* under its native LexA-regulated promoter at the *fhuB* locus. This system was introduced into both *wildtype* and Δ*seqA* strains, enabling direct visualization and quantification of SOS activation at both the single-cell (microscopy) and population (flow cytometry) levels. To quantify DSB formation, cells were treated with ciprofloxacin at sub-MIC and MIC concentrations (1, 5, 10, 15, and 25 ng/mL). We selected 1 ng/mL as the lowest sub-MIC reference, as Δ*recA* strains (defective in DSB repair) could not survive at this concentration (data not shown), indicating that DSBs occur even at such low doses. Fluorescence microscopy revealed a gradual formation of MuGam-GFP foci in *wildtype* cells after 2 hours of exposure to 5 ng/mL ciprofloxacin, confirming DSB induction at sub-MIC concentrations. However, cells efficiently repaired these breaks as no significant reduction in CFU was observed at this dose (Figure 2E). At higher concentrations (10, 15, and 25 ng/mL), foci formation increased progressively, consistent with increased DSB formation, compromised repair, and reduced CFU counts (Figure 2E,F,G). At the population level, flow cytometry analysis of ~100,000 cells per sample confirmed these results. At 5 ng/mL, cells with MuGam-GFP foci increased from 2.74 ± 0.15% to 22.36 ± 0.8% after 2 and 4 hours, respectively. At 15 ng/mL, the corresponding values were 21.33 ± 2.44% and 57.1 ± 0.78% (Figure 2A–D).

In contrast, Δ*seqA* strains required a higher ciprofloxacin concentration (15 ng/mL) to exhibit MuGam-GFP foci accumulation comparable to the *wildtype* response at 5 ng/mL (Figure 2E). Single-cell microscopy (n = 800 cells per sample) showed that after 2 hours of ciprofloxacin treatment at 5, 10, 15 and 25 ng/mL, MuGam-GFP foci were present in 5.9 ± 0.6%, 7.43 ± 0.82%, 16.86 ± 1.25%, and 72.38 ± 5.23% of Δ*seqA* cells, respectively. After 4 hours, these percentages increased to 6.23 ± 1.24%, 9.32 ± 0.96%, 28.43 ± 2.76%, and 76.28 ± 6.37%. Notably, the proportion of MuGam-GFP-positive Δ*seqA* cells at 15–25 ng/mL matched the *wildtype* response at 5–10 ng/mL, indicating greater resistance to ciprofloxacin (Figure 2F–G). Flow cytometry supported these findings, revealing that Δ*seqA* strains treated with 10, 15, and 25 ng/mL ciprofloxacin had 0.67 ± 0.06%, 4.5 ± 1.724%, and 29 ± 3.23% MuGam-GFP-positive cells after 2 hours, rising to 1.71 ± 0.17%, 16 ± 3.09%, and 57 ± 0.89% after 4 hours (Figure 2A–D). Similar results were obtained when cells were treated continuously for 8 hours at different concentrations (Figure S4A–D). All flow-cytometry analyses were gated using the 0 h untreated (0 ng/mL ciprofloxacin) sample of each strain to define the GFP-negative population. For data representation, fluorescence intensities and GFP-positive cell percentages were normalized within each strain, such that *wildtype* samples were compared to their untreated *wildtype* control and Δ*seqA* samples to their untreated Δ*seqA* control. This per-strain normalization accounts for minor baseline differences in GFP fluorescence that may arise from physiological variation such as cell size or reporter background. The dotted vertical line in each histogram indicates the mean fluorescence intensity (MFI) of the untreated control, providing a visual reference for treatment-induced GFP shifts. All samples were acquired using identical cytometer settings to ensure valid comparisons between *wildtype* and Δ*seqA* populations. Collectively, these results suggested that Δ*seqA* mutants have fewer double-strand breaks (DSBs) at the *wildtype* minimum inhibitory concentration (MIC) of 15 ng/mL, requiring a higher ciprofloxacin dose (25 ng/mL) to induce comparable DSB levels, consistent with an approximately 1.5-fold MIC increase.

To independently validate that the MuGam-GFP reporter faithfully detects DNA double-strand breaks in our experimental system, we included mitomycin C (MMC) as a positive control. MMC is a well-characterized DNA crosslinking agent that generates replication-dependent DSBs through fork collapse [9],[36],[37]. *Wildtype* and Δ*seqA* cells carrying the MuGam-GFP reporter were treated with 2 µg/mL MMC and imaged after 2 and 4 hours, alongside untreated controls. As expected, both strains showed robust induction of MuGam-GFP foci following MMC exposure, with a clear increase in the proportion of cells containing discrete fluorescent foci relative to untreated conditions (Figure S5). Quantification confirmed a strong, time-dependent elevation in DSB-associated signal in both strains. These results verified that the MuGam-GFP system is fully responsive in the Δ*seqA* mutant and confirmed that the reduced DSB signal observed under ciprofloxacin exposure reflects genuine biological differences rather than impaired reporter functions. Collectively, these results suggested that Δ*seqA* mutants have fewer double-strand breaks (DSBs) at the *wildtype* minimum inhibitory concentration (MIC) of 15 ng/mL, requiring a higher ciprofloxacin dose (25 ng/mL) to induce comparable DSB levels, consistent with an approximately 1.5-fold MIC increase.

**Figure 3.**
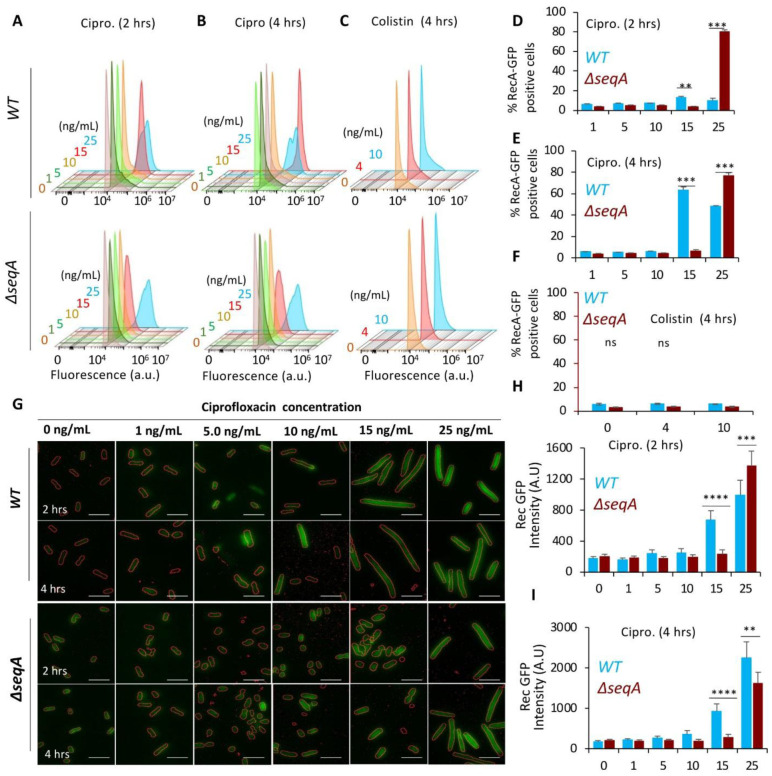
Impaired SOS response in Δ*seqA* mutants following ciprofloxacin treatment. (A–C) Flow cytometry histograms showing RecA-GFP fluorescence in *wildtype* and Δ*seqA* strains treated with ciprofloxacin for 2 and 4 hours and colistin for 4 hours. (D–F) Quantification of RecA-GFP–positive cells from a population of 100,000 cells, following treatment with ciprofloxacin and colistin. (G) Representative fluorescence microscopy images of *wildtype* and Δ*seqA* cells expressing RecA-GFP (green) after 2- and 4-hours of ciprofloxacin treatment. (H, I) Quantification of RecA-GFP fluorescence intensity under microscope at 2- and 4-hours post-treatment. Data represent mean ± SD from three independent experiments. Scale bar represent 2 µm. *, **, and *** represent the significance p-value of 0.01, 0.001, and 0.0001, respectively. Statistical significance was determined by Student's t-test (***p < 0.001).

As SOS induction is a direct downstream response to persistent DNA double-strand breaks, we next examined whether the reduced DSB formation in Δ*seqA* corresponded to altered activation of the SOS pathway. To test this, we quantified RecA-GFP expression in *wildtype* and Δ*seqA* strains after ciprofloxacin treatment. Flow cytometry analysis of RecA-GFP positive cells corroborated the MuGam-GFP data, where 15 ng/mL ciprofloxacin, 13.06 ±1.04% and 63.3 ± 2.78% of *wildtype* cells were GFP-positive after 2 and 4 hours, respectively, compared to only 3.63 ±1.04% and 6.74 ± 0.9% of Δ*seqA*, respectively. At the MIC of Δ*seqA* (25 ng/mL), GFP-positive cells increased to 76 ± 3.09% and 80 ± 1.7% after 2 and 4 hours, respectively (Figure 3A-B, D-E). The similar trend was observed even if the cells were continuously treated up to 8 hours with different concentrations of ciprofloxacin (Figure S6A–D). At the single-cell level, RecA-GFP intensity in *wildtype* cells treated with 15 ng/mL ciprofloxacin was comparable to that in Δ*seqA* strains treated with 25 ng/mL (Figure 3G–I).

To confirm that the observed SOS induction was specific to DNA-damaging conditions and not a general response to antibiotic stress, *wildtype* and Δ*seqA* strains were treated with colistin at its MIC and MBC concentrations (1 and 4 µg/mL, respectively). Colistin, which targets the bacterial outer membrane and does not directly induce DNA damage, served as a negative control [38]. Neither strain showed any appreciable increase in RecA-GFP fluorescence, indicating that the SOS response was not activated under these conditions (Figure 3C, F)

Collectively, these results demonstrated that, compared to *wildtype* cells (MIC: 15 ng/mL), Δ*seqA* mutants require higher ciprofloxacin concentrations (25 ng/mL) to reach equivalent levels of DSB accumulation (MuGam-GFP foci) and SOS induction (RecA-GFP). This explains the observed ~1.5-fold increase in MIC in Δ*seqA* strains. However, reduced DNA damage and delayed SOS activation alone could not fully account for the enhanced survival of Δ*seqA* at fluoroquinolone concentrations lethal to *wildtype* cells. This prompted us to examine whether additional global stress pathways, particularly the RpoS-mediated general stress response, contribute to the resistance phenotype.

**Figure 4.**
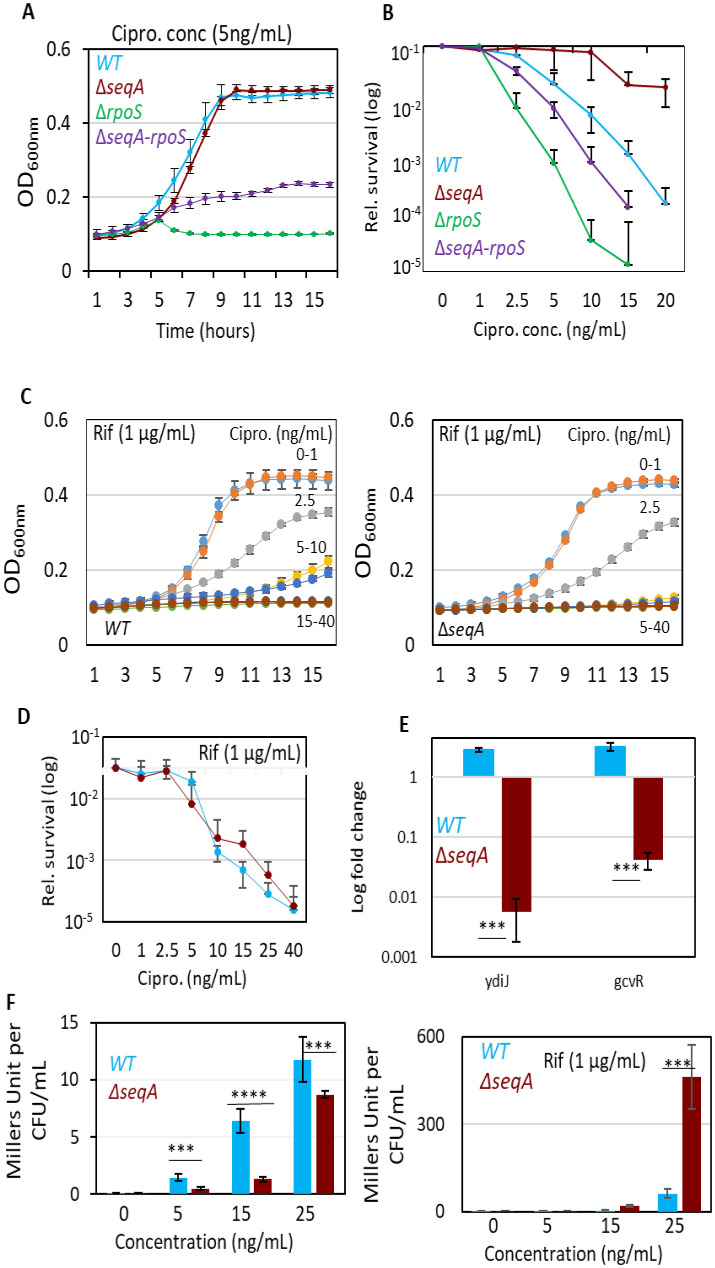
Loss of RpoS or transcriptional inhibition sensitizes ΔseqA mutants to ciprofloxacin. (A) Growth kinetics of *wildtype* (*WT*), Δ*seqA*, Δ*rpoS*, and Δ*seqA-rpoS* strains in M9-glucose medium in the presence of ciprofloxacin (5 ng/mL). OD₆₀₀ was monitored over 15 hours. (B) Corresponding relative CFU counts after 15 hours of ciprofloxacin exposure, highlighting the intermediate sensitivity of the Δ*seqA-rpoS* double mutant relative to Δ*seqA* (resistant) and Δ*rpoS* (sensitive) single mutants. (C) Growth curves of *WT* and Δ*seqA* strains in the presence of ciprofloxacin gradient (0–40 ng/mL) with sub-MIC rifampicin (1 µg/mL). (D) Relative survival measured by CFU enumeration and spot dilution assay for the same conditions as in (C), showing that sub-MIC rifampicin reduces ciprofloxacin resistance in Δ*seqA* cells. (E) RT-qPCR analysis of supercoiling-sensitive genes (*ydiJ* and *gcvR*) following 2-hour treatment with ciprofloxacin in mid-log phase. Expression values are normalized to *lacI* and plotted as fold change. Both genes show marked repression in the Δ*seqA* strain, consistent with a shift toward a more relaxed DNA supercoiling state. (F) β-galactosidase activity of the *sulA-lacZ* reporter (expressed as Miller units per CFU) in *WT* and Δ*seqA* strains treated with ciprofloxacin alone (left) or ciprofloxacin plus sub-MIC rifampicin (right). Data represent mean ±SD of at least three independent biological replicates. Statistical significance was determined by Student's t-test (***p < 0.001).

### 3.3. Low-level resistance to Ciprofloxacin in ΔseqA strains is reversed in RpoS deletion and treatment with sub-MIC concentration of rifampicin

Given that the low-level resistance to ciprofloxacin in Δ*seqA* strains arises from reduced DNA double-strand breaks formation and attenuated SOS induction, we next investigated whether this phenotype requires activation of the general stress response. RpoS (σ^S^) regulates numerous genes linked to oxidative stress defense, DNA repair, and stationary-phase survival, making it a strong candidate for contributing to the enhanced ciprofloxacin resistance observed in Δ*seqA* [39].

To assess this, we constructed Δ*rpoS* and Δ*seqA-rpoS* mutants and compared their ciprofloxacin susceptibility to *wildtype* and Δ*seqA* strains. As discussed, MIC analysis revealed that *wildtype* cells exhibited a ciprofloxacin MIC of 15 ng/mL, whereas the Δ*seqA* strain displayed an increased MIC of 25 ng/mL. In contrast, the Δ*rpoS* mutant was hypersensitive with an MIC of only 5 ng/mL. Importantly, the Δ*seqA-rpoS* double mutant showed an intermediate MIC of 10 ng/mL, higher than Δ*rpoS* but markedly reduced compared to Δ*seqA* (Figure 4A and Figure S7A). MBC analysis paralleled these trends that Δ*seqA* exhibited an MBC of 80 ng/mL compared to 40 ng/mL for *wildtype*, while the Δ*rpoS* and Δ*seqA-rpoS* mutants showed reduced MBCs of 40 ng/mL (Figure 4B and Figure S7B). These findings indicated that RpoS activity is epistatic to SeqA and required for the expression of the ciprofloxacin resistance phenotype.

To determine whether Δ*seqA* resistance is associated with elevated RpoS induction, we quantified *rpoS* transcript levels by RT-qPCR. No significant differences in *rpoS* expression were detected between *wildtype* and Δ*seqA*, either in untreated conditions or following ciprofloxacin exposure at MIC levels (Figure S7D). These data suggested that basal RpoS activity rather than its transcriptional upregulation is necessary for the Δ*seqA* resistance phenotype. Because the resistance phenotype depended on basal RpoS activity and could not be explained solely by attenuated SOS induction, we next tested whether perturbing global transcription would influence Δ*seqA* mediated resistance. Cells were co-treated with sub-MIC rifampicin (1 µg/mL), a concentration that minimally affected *wildtype* growth but partially inhibited RNA-Polymerase (RNAP) function. Under these conditions, Δ*seqA* cells exhibited a marked reduction in ciprofloxacin resistance as MIC decreased from 25 ng/mL to 10 ng/mL, and the MBC dropped from 80 ng/mL to 40 ng/mL; values comparable to *wildtype* (Figure 4C,D and Figure S7C). In contrast, rifampicin co-treatment had little effect on *wildtype* susceptibility. Furthermore, β-galactosidase assays of *sulA-lacZ* activity showed that SOS induction remained suppressed in Δ*seqA*, but the combined transcriptional inhibition and loss of SeqA had significantly increased SOS induction compared to *wildtype* (Figure 4F).

Together, these results demonstrated that the low-level ciprofloxacin resistance of Δ*seqA* strains is transcription-dependent and requires basal RpoS activity. Deletion of *rpoS* or transcriptional inhibition by rifampicin reverses the resistant phenotype, highlighting the interplay between SeqA, transcriptional adaptation, and stress response pathways in shaping bacterial survival under fluoroquinolone stress.

To determine whether loss of SeqA affects global DNA topology during fluoroquinolone exposure, we examined the expression of two well-characterized supercoiling-sensitive genes (SSGs), *ydiJ* and *gcvR*, both classified as relaxation-repressed genes in the comprehensive supercoiling transcriptome map [40]. Mid-log cultures of *wildtype* and Δ*seqA* strains were treated with 15 ng/mL ciprofloxacin for 2 hours, and transcript levels were quantified by RT-qPCR. *ydiJ* and *gcvR* showed marked downregulation in the Δ*seqA* strain compared with *wildtype* (Figure 4E). Because relaxation-repressed genes are transcriptionally silenced when chromosomal DNA becomes less negatively supercoiled, this pattern indicates that Δ*seqA* cells experience a relative shift toward a relaxed topological state during ciprofloxacin stress. Such relaxation is mechanistically relevant, as fluoroquinolones stabilize gyrase–DNA cleavage complexes most efficiently on highly negatively supercoiled DNA [41],[42]. A reduction in negative supercoiling would therefore be expected to decrease cleavage-complex formation, providing a plausible topological basis for the lower DSB burden and higher MIC observed in the Δ*seqA* mutant.

## 4. Discussion

Our findings showed that loss of SeqA leads to a reproducible, low-level increase in fluoroquinolone resistance (LLQR) in *E. coli*, a phenotype tightly linked to changes in replication dynamics and global genome organization. This is consistent with the well-established multifunctional role of SeqA in coordinating replication initiation, stabilizing newly replicated strands, and maintaining local DNA supercoiling. Because fluoroquinolones induce DNA damage by trapping topoisomerase-DNA cleavage complexes and promoting double-strand breaks, the LLQR phenotype of the Δ*seqA* mutant needs to be interpreted in the broader context of chromosome dynamics.

### 4.1. SeqA loss reduces DSB formation and attenuates SOS activation

At ciprofloxacin concentrations corresponding to the *wildtype* MIC, the Δ*seqA* strain accumulates fewer DSB foci, as indicated by reduced MuGam-GFP signal, a phage-derived marker of double-stranded DNA ends. Notably, this reduced susceptibility was specific to fluoroquinolones, as no MIC or MBC changes were observed for β-lactams, aminoglycosides, or membrane-active agents, indicating a mechanistic link to replication-associated DNA damage rather than broad-spectrum stress resistance. The fluoroquinolone-specific reduction in DSBs in Δ*seqA* can be explained by an increased asynchronous initiation that results in fewer active replication forks available to collide with drug-stabilized gyrase-DNA cleavage complexes, thereby reducing fork-collapse-driven DSB formation [43]. In addition, SeqA is known to influence chromosome topology [44]. To assess whether loss of SeqA alters DNA supercoiling during fluoroquinolone stress, we examined the expression of supercoiling-sensitive genes identified in a genome-wide study [40]. Consistent with this, our RT-qPCR data showed downregulation of two supercoiling sensitive genes, *ydiJ* and *gcvR*, in the Δ*seqA* strain. Thus, loss of SeqA will result in relaxed chromosomes, which makes it a poor substrate for fluoroquinolones, preferentially binding to negatively supercoiled DNA [41],[42]. Therefore, in the Δ*seqA* strain, reduced replication-fork availability and a relaxed chromosomal topology limit the efficacy of fluoroquinolone-mediated DNA damage, resulting in an attenuated SOS response. A similar phenomenon has been observed in mutants with altered DNA topology or replication initiation timing which exhibit modified sensitivity to quinolones [45]. While we focused on fluoroquinolone-induced DNA damage, it is well established that the absence of SeqA can cause replication fork instability and spontaneous DNA breaks under normal growth conditions [10]. However, in our assays, baseline RecA-GFP and MuGam-GFP fluorescence in untreated cultures did not differ appreciably between *wildtype* and Δ*seqA* strains. One possible explanation is that all experiments were performed in M9-glucose medium, where the slower growth rate and reduced replication initiation frequency minimized spontaneous replication stress. This likely prevented the manifestation of baseline DNA damage that has been reported under faster growth conditions in rich media. Together, these observations suggest that the differences in DNA damage and SOS induction between *wildtype* and Δ*seqA* strains primarily arise under fluoroquinolone stress rather than from inherent instability of the mutant.

### 4.2. RpoS dependence links chromosome disorganization to stress physiology

Besides its dependence on the SOS response, the LLQR phenotype in the Δ*seqA* strain also required basal RpoS activity. The expression of *rpoS* did not change even at 1.5× MIC, implying that replication defects and altered DNA supercoiling in Δ*seqA* are somehow linked to RpoS-mediated stress adaptation. The dependence of LLQR in Δ*seqA* strains on global transcriptional changes was further supported by rifampicin co-treatment, which abolished LLQR in this strain. While we did not dissect the specific downstream genes regulated by RpoS that contribute to this phenotype, our genetic and phenotypic data established that RpoS activity is required for Δ*seqA* associated fluoroquinolone resistance. Because RpoS controls multiple stress-protective pathways, including oxidative defense, DNA repair, and redox homeostasis [20],[46], we propose that this effect likely results from broad transcriptional reprogramming rather than the action of a single effector gene. Future transcriptomic or proteomic studies will be necessary to define the precise RpoS-dependent factors involved. This finding aligns with established literature showing that basal RpoS activity promotes antibiotic resistance under nutrient limitation by enhancing redox homeostasis and DNA damage repair capacity [20]. However, a functional connection to SeqA-dependent replication control had not been established. Our results also demonstrate that the resistant phenotype of the Δ*seqA* strain is dependent on transcription.

### 4.3. Model for SeqA-dependent LLQR

**Figure 5.**
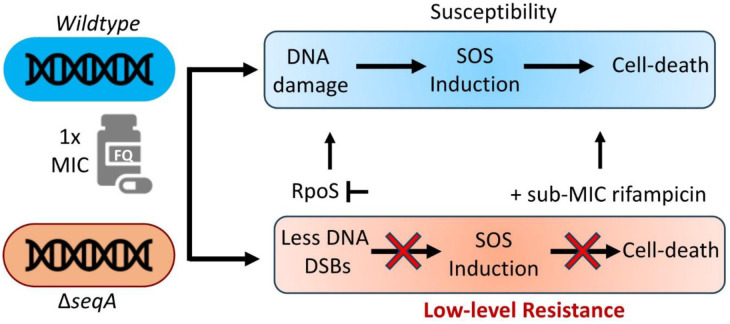
Schematic model illustrating SeqA-mediated low-level resistance to fluoroquinolones (FQs) in *Escherichia coli*. Loss of SeqA reduces ciprofloxacin-induced DNA double-strand breaks (DSBs) and attenuates SOS activation, thereby enabling survival under conditions that are lethal to *wildtype* cells. Deletion of *rpoS* or treatment with sub-MIC rifampicin abolishes this resistance and restores ciprofloxacin sensitivity. This low-level resistance phenotype depends on RpoS-mediated transcriptional reprogramming. This low-level resistance phenotype depends on basal RpoS activity and global transcriptional modulation that sustains protective stress responses under genotoxic stress.

The modest increase in MIC/MBC seen in the Δ*seqA* strain falls within the LLQR range. However, it provides an important clue about how microbes can subtly alter chromosome organization, DNA topology, and downstream gene expression to modulate the SOS response and increase tolerance to antibiotics. Beyond the immediate resistance phenotype, low-level quinolone resistance (LLQR) is often considered an early adaptive state that can precede the evolution of high-level resistance. Future work involving serial passaging and genome sequencing of Δ*seqA* strains under sub-MIC fluoroquinolone exposure could reveal whether altered replication dynamics promote the accumulation of resistance conferring mutations. It is also important to consider that all experiments in this study were performed in M9–glucose minimal medium, where slower growth and fewer replication cycles inherently limit mutation fixation and adaptive evolution. These growth conditions likely constrained the emergence of high-level resistance within the experimental timeframe [47]–[49]. Our results support a unified model for SeqA-dependent LLQR (Figure 5). Loss of SeqA decreases replication fork density, reducing the probability that active forks will collide with fluoroquinolone-stabilized topoisomerase-DNA cleavage complexes. Furthermore, the Δ*seqA* nucleoid becomes more relaxed, lowering gyrase activity and reducing the efficiency of drug-induced double-strand break formation. Research has shown that Δ*seqA* mutants exhibit hypersensitivity to replication inhibitors such as UV, Azidothymidine (AZT), and hydroxyurea, and that this phenotype is strongly dependent on growth medium richness because high replication-initiation frequency in rich media destabilizes replication forks [50]. Our findings are consistent with this model, as Δ*seqA* cells display pronounced growth defects and reduced CFU counts in LB and Mueller–Hinton media even in the absence of antibiotics, whereas growth is comparable to *wildtype* in M9–glucose (Figure S1). These chromosome-level alterations appear to activate RpoS-dependent transcriptional programs that enhance oxidative and metabolic resilience, collectively enabling cells to better withstand genotoxic stress. The specificity of the MIC increase in the Δ*seqA* strain is restricted to DNA-damaging antibiotics, implying that SeqA functions not only as a regulator of replication initiation but also as a determinant of fluoroquinolone susceptibility through its combined influence on DNA topology, replication dynamics, and global stress physiology. Overall, our findings highlight how chromosome organization and the interplay between replication, topology, and stress responses shape bacterial resistance to genotoxic antibiotics.

## 5. Conclusion

Our findings reveal that, in the absence of SeqA, *E. coli* exhibit low-level resistance to fluoroquinolones by exploiting the following strategies: (a) Reducing the formation of lethal DNA double-strand breaks, thereby providing physical protection, (b) inducing attenuated activation of the SOS response, effectively delaying damage signaling, and (c) relying on transcriptional modulation and basal RpoS activity to sustain protective stress responses. Taken together, these results redefine SeqA as a central coordinator that links replication control, chromosome organization, and global gene regulation in shaping antibiotic susceptibility. Such non-mutational resistance strategies may promote bacterial persistence during treatment, potentially contributing to recurrent or chronic infections. Targeting these adaptive mechanisms could provide new avenues to enhance the effectiveness of existing antibiotics.


