# Supplementary material for: Loss of SeqA confers low-level fluoroquinolone resistance through transcriptional reprogramming and RpoS dependence in E. coli
Source: AIMS Microbiol. 2025 Dec 24;11(4):1079–100. doi: 10.3934/microbiol.2025047 (PMC12782930; doi:10.3934/microbiol.2025047)
Supplement: Supplementary file 1 [file microbiol-11-04-047-s001.pdf]

AIMS Microbiology

AIMS Microbiology, 11(4): 1079–1100.
DOI: 10.3934/microbiol.2025047
Received: 26 August 2025
Revised: 13 December 2025
Accepted: 18 December 2025
Published: 24 December 2025

http://www.aimspress.com/journal/microbiology

Research article

Loss of SeqA confers low-level fluoroquinolone resistance through transcriptional reprogramming and RpoS dependence in *E. coli*

Amir Faraz, Nuha Abeer Khan, Deepak Kumar Singh, Hamna Syed and Mohan C. Joshi*

Multidisciplinary Centre for Advanced Research and Studies (MCARS), Jamia Millia Islamia, New Delhi, India-110025

* **Correspondence:** Email: mjoshi@jmi.ac.in.

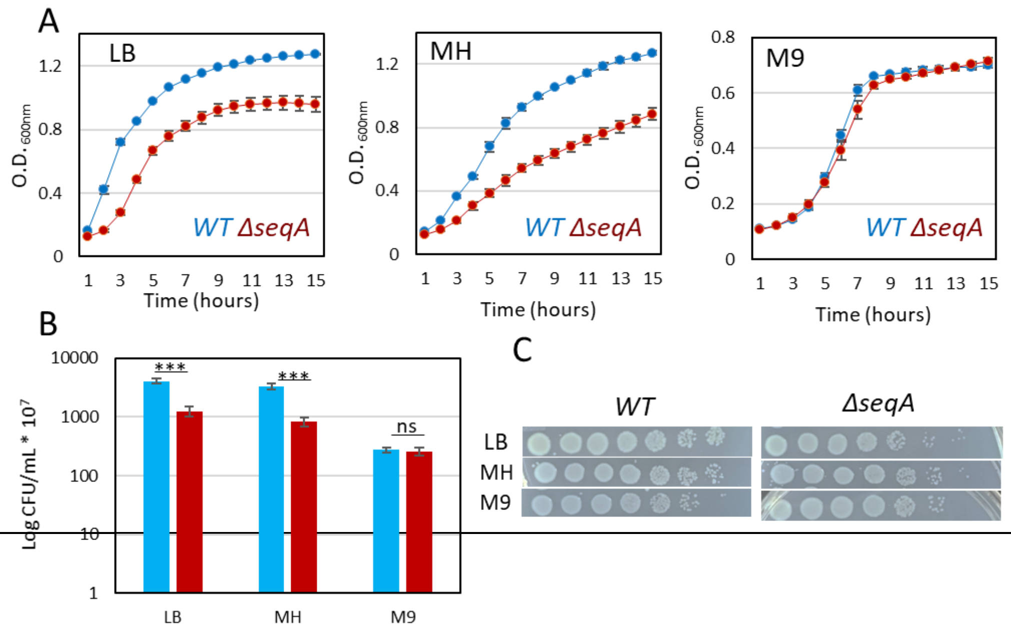

Figure S1. Growth and viability of *wildtype* and ΔseqA in LB, Mueller–Hinton, and M9-glucose media. (A) Growth curves of *wildtype E. coli* MG1655 and the Δ*seqA* mutant grown in LB, Mueller–Hinton (MH), and M9-glucose media at 37°C with shaking. Δ*seqA* exhibits pronounced growth defects in LB and MH, characterized by reduced optical density and delayed exponential-phase entry, whereas both strains show comparable growth in M9-glucose. (B) Viable cell counts (Log CFU/mL) for *wildtype* and Δ*seqA* after growth in LB, MH, and M9–glucose. Consistent with the growth curves, Δ*seqA* shows markedly lower CFU in LB and MH, indicating impaired viability under fast growth conditions. In contrast, CFU values in M9–glucose are comparable between strains. (C) Spot dilution assays showing survival of *WT* and Δ*seqA* strains on grown in LB, MH and M9-glucose media. Plates were incubated for 24 h at 37 °C. Data points are average of at least 3 independent experiments. Error bars represent standard error.

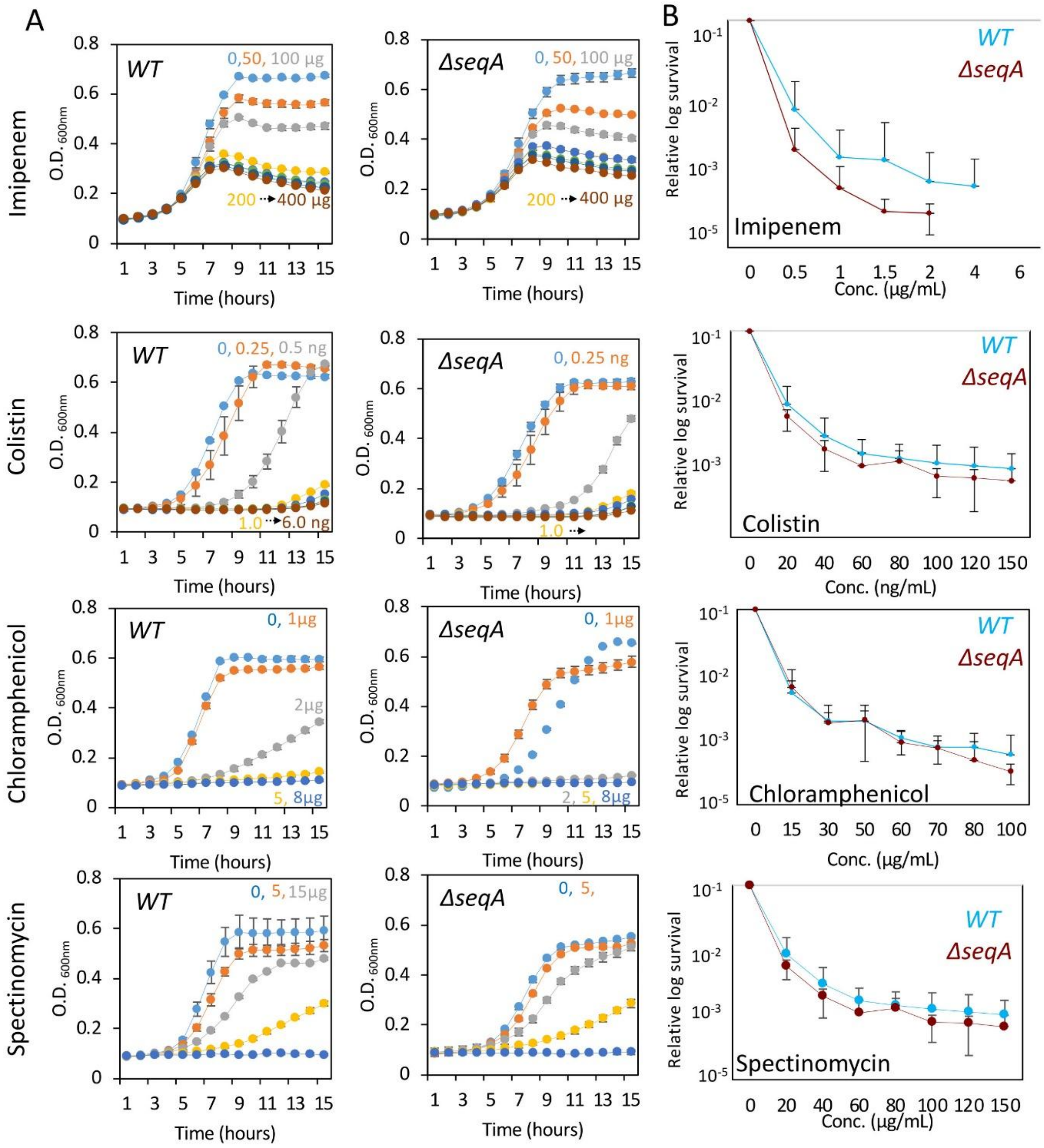

Figure S2. Growth and survival of *wildtype* and Δ*seqA* strains in the presence of non-fluoroquinolone antibiotics. (A) Growth curves of *wildtype* (*WT*) and Δ*seqA* strains treated with increasing concentrations (in μg/mL) of Imipenem, Colistin, Chloramphenicol and Spectinomycin in M9-glucose minimal medium. OD_{600} was monitored over time using a microplate reader. The Δ*seqA* mutant showed increased sensitivity to Imipenem, comparable growth inhibition to *WT* under Colistin and Spectinomycin treatment, and moderately reduced growth in the presence of Chloramphenicol, particularly at higher concentrations. (B) Corresponding survival assays after 15 hours of antibiotic exposure, expressed as relative log survival compared to untreated controls. The Δ*seqA* strain exhibited lower survival under Imipenem and high-dose Chloramphenicol treatment, whereas both strains displayed similar susceptibility to Colistin and Spectinomycin. Data

points are average of at least 3 independent biological replicates. Error bars represent standard error.

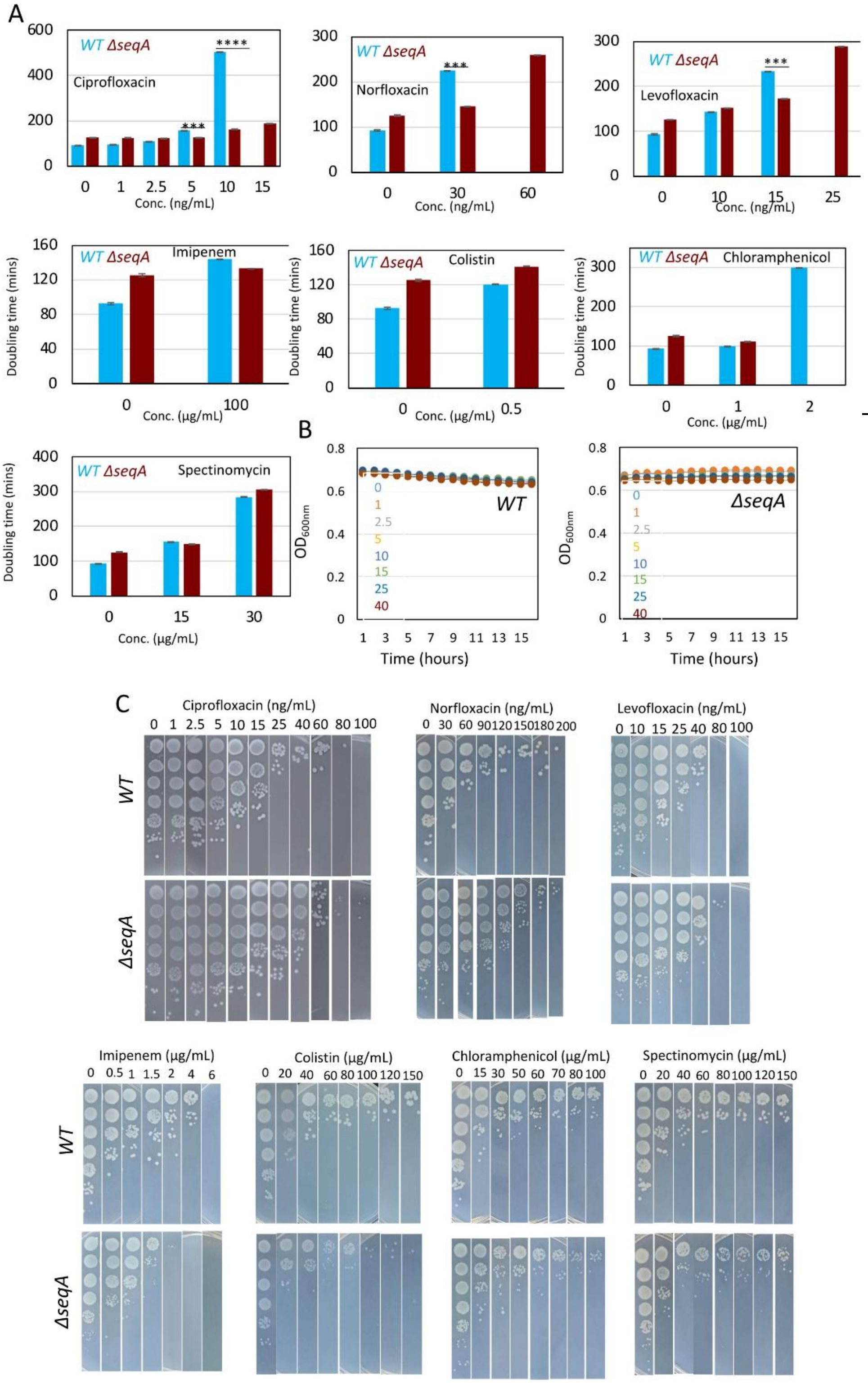

Figure S3. Growth dynamics and replication-dependent antibiotic response of *wildtype* and Δ*seqA* strains. (A) Doubling times of *wildtype* (WT) and Δ*seqA* strains calculated from the exponential phase of the same growth curves presented in SI Fig. 1. Doubling time was

determined in M9-glucose medium supplemented with increasing concentrations (in µg/mL) of ciprofloxacin, norfloxacin, levofloxacin, imipenem, colistin, chloramphenicol, and spectinomycin. An increased doubling time indicates antibiotic-induced stress or slowed replication. (B) Stationary-phase ciprofloxacin treatment. Cultures of *WT* and Δ*seqA* strains grown to saturation in M9-glucose were treated with ciprofloxacin (0-40ng/mL) to determine whether the resistance phenotype depends on active replication. Both strains exhibited no susceptibility under stationary-phase conditions, confirming that the increased ciprofloxacin resistance of Δ*seqA* is replication dependent and manifests only during active growth. (C) Spot dilution assays showing survival of *WT* and Δ*seqA* strains on M9-glucose agar plates grown in the antibiotic concentrations used in MIC assays. Plates were incubated for 24 h at 37 °C. Data points are average of at least 3 biological replicates. Error bars represent standard error.

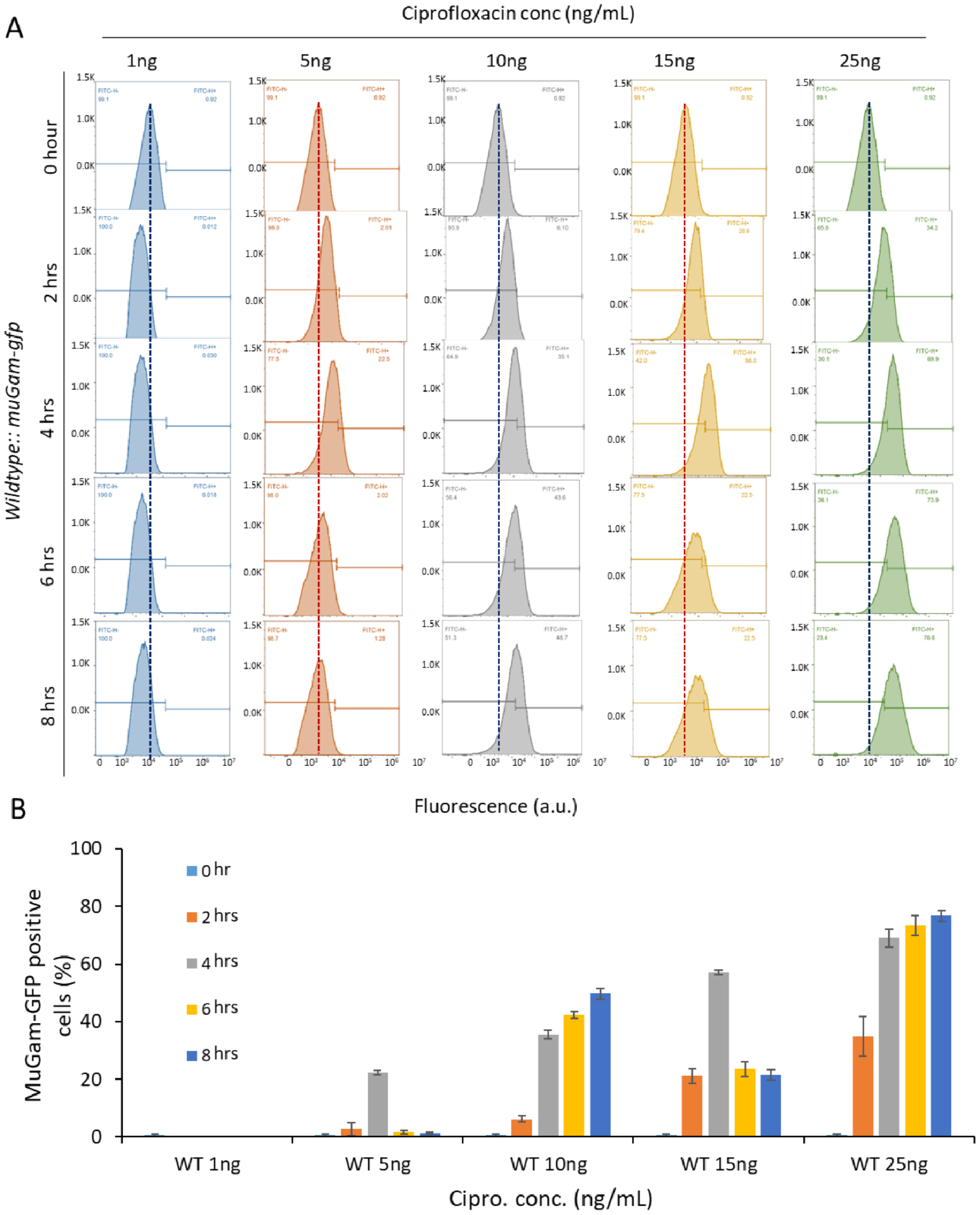
A
Ciprofloxacin conc (ng/mL)
1ng
5ng
10ng
15ng
25ng
0 hour
2 hrs
4 hrs
6 hrs
8 hrs
Wildtype:: muGam-gfp
Fluorescence (a.u.)
B
MuGam-GFP positive cells (%)
0 hr
2 hrs
4 hrs
6 hrs
8 hrs
WT 1ng
WT 5ng
WT 10ng
WT 15ng
WT 25ng
Cipro. conc. (ng/mL)

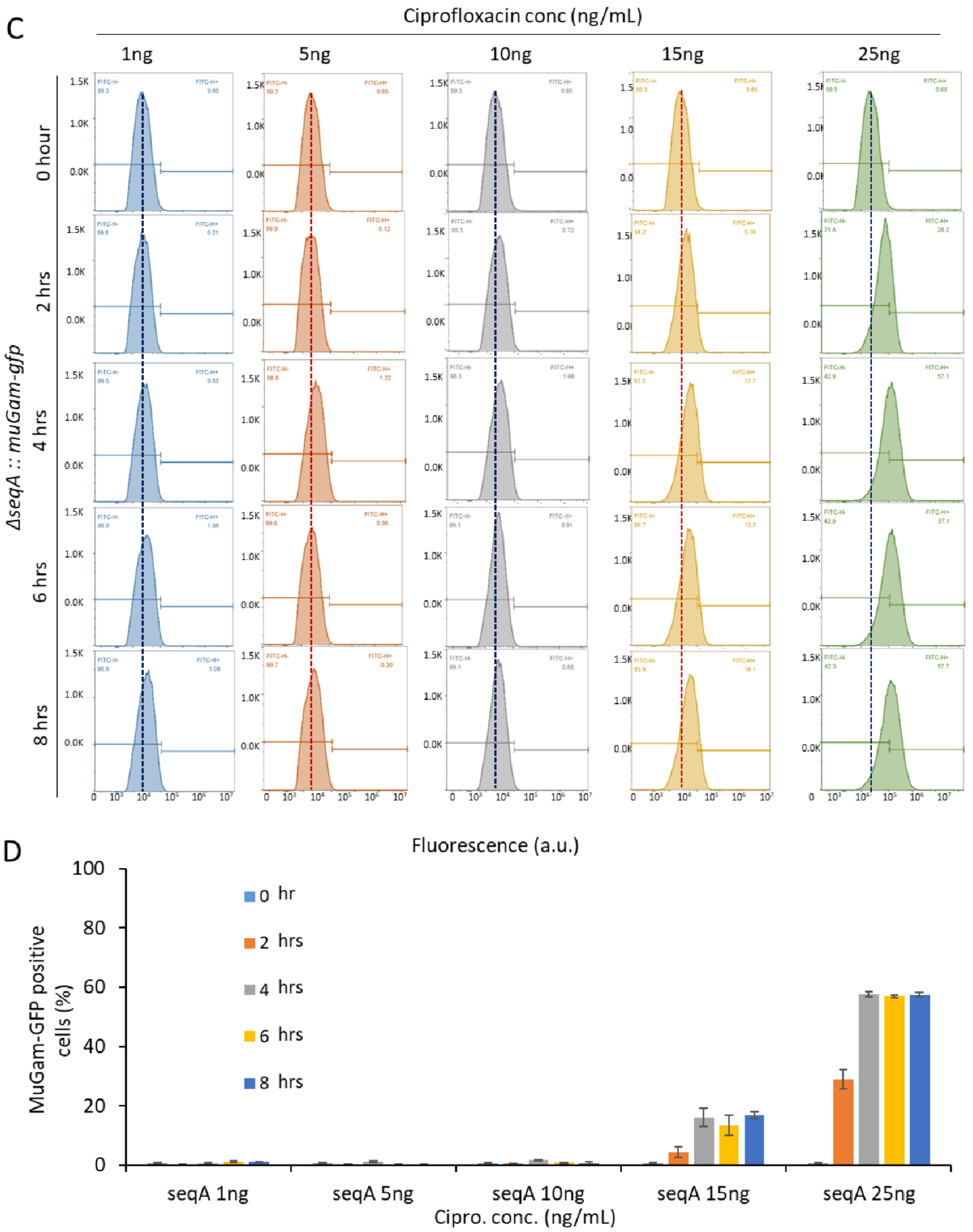

Figure S4. Flow cytometry analysis of DNA double-strand breaks (muGAM-GFP expression) in *wildtype* and *ΔseqA* strains after 8-Hour ciprofloxacin treatment. Histograms of MuGam-GFP fluorescence intensity of (A) *wildtype* and (C) *ΔseqA* strains in presence of varying concentration of ciprofloxacin treated for 8 hrs. (B, D) quantification of mean GFP fluorescence intensity across biological triplicates. Data are shown as mean ± SD. At least 50,000 events were collected per sample. Note: Flow cytometry data were gated using the untreated (0 h) sample of each strain as the reference.

The dotted vertical line indicates the mean fluorescence intensity (MFI) of the untreated control, representing the baseline for GFP-negative cells. Shifts to the right of this line indicate increased MuGam-GFP or RecA-GFP signal relative to baseline. All samples were acquired using identical instrument settings, allowing valid comparison of fluorescence induction between strains.

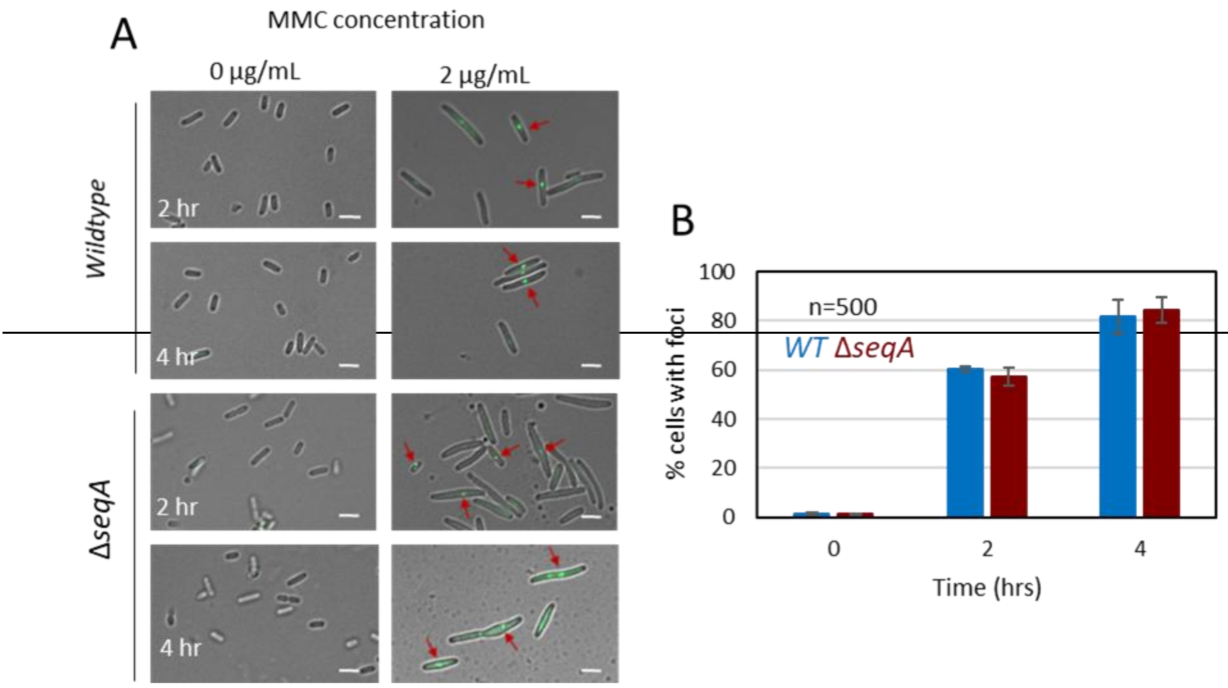

Figure S5. Validation of MuGam-GFP reporter using mitomycin C. (A) Representative fluorescence microscopy images of *wildtype* (*WT*) and Δ*seqA* cells expressing MuGam-GFP, untreated or treated with 2 µg/mL mitomycin C (MMC) for 2 h and 4 hr. Arrows indicate MuGam-GFP foci corresponding to DNA double-strand breaks. Scale bar: 2 µm. (B) Quantification of the percentage of MuGam-GFP–positive cells under the indicated conditions. MMC treatment induces strong MuGam-GFP foci in both *WT* and Δ*seqA* backgrounds, confirming that the reporter detects DSBs independently of ciprofloxacin exposure. Data represent mean ± SD from at least three fields of view.

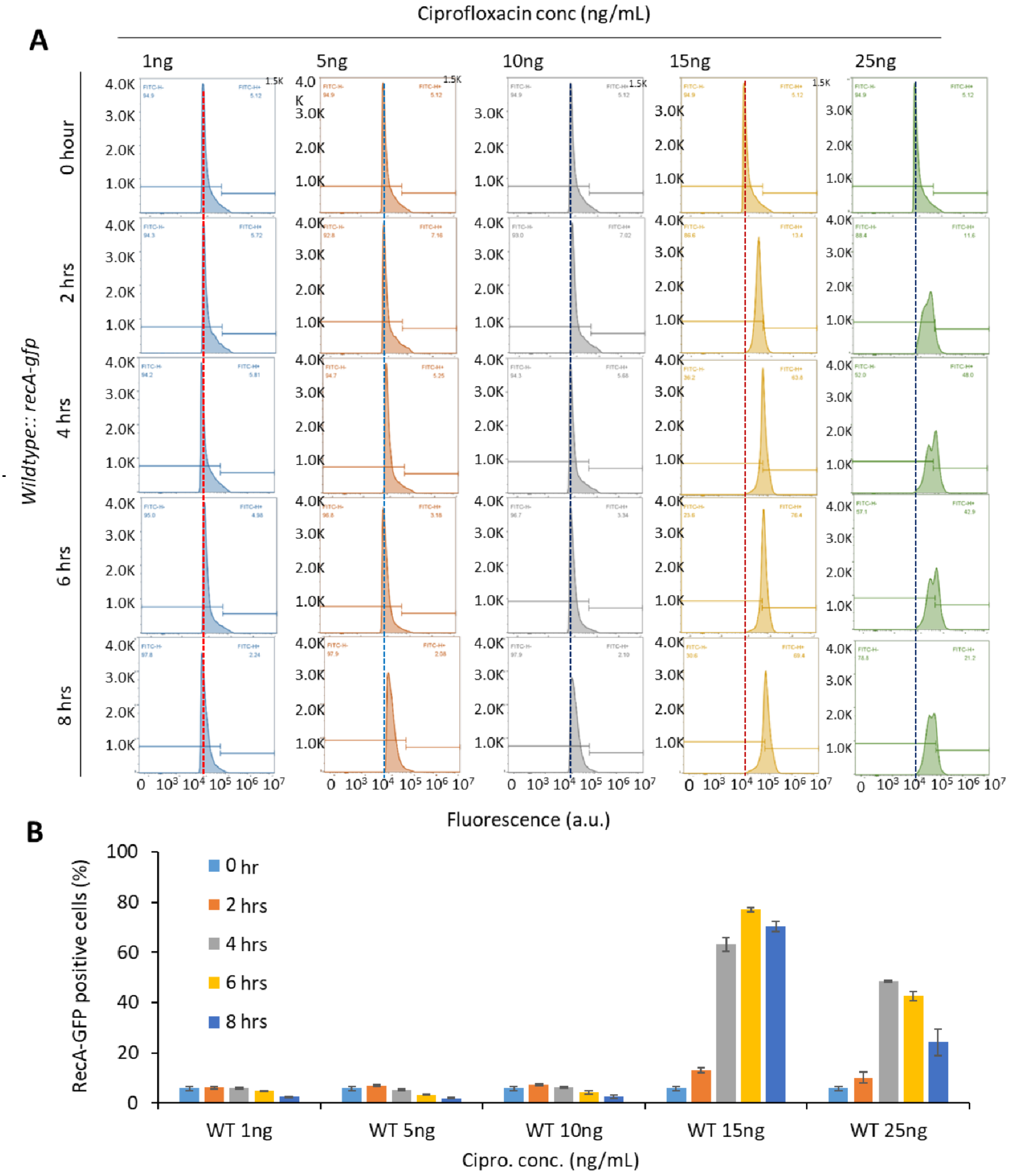
A
Ciprofloxacin conc (ng/mL)
1ng
5ng
10ng
15ng
25ng
0 hour
2 hrs
4 hrs
6 hrs
8 hrs
Wildtype:: recA-gfp
Fluorescence (a.u.)
B
RecA-GFP positive cells (%)
0 hr
2 hrs
4 hrs
6 hrs
8 hrs
WT 1ng
WT 5ng
WT 10ng
WT 15ng
WT 25ng
Cipro. conc. (ng/mL)

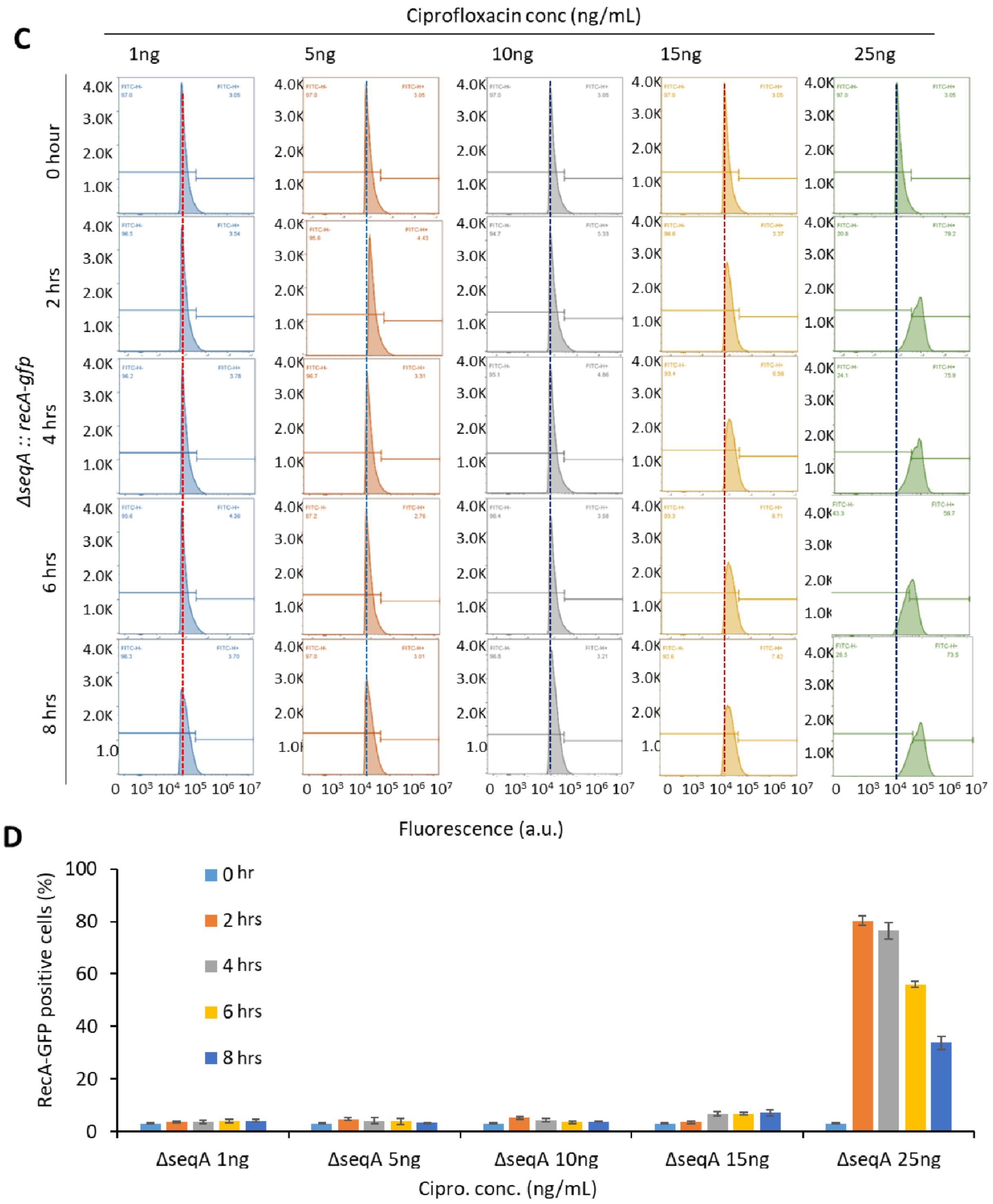

Figure S6. Flow cytometry analysis and quantification of SOS Response (RecA-GFP expression) in *wildtype* and *ΔseqA* strains after 8-hour ciprofloxacin exposure. Histograms of RecA-GFP fluorescence intensity of (A) *wildtype* and (C) *ΔseqA* strains in presence of varying concentration of ciprofloxacin treated for 8 hrs. (B, D) quantification of mean GFP fluorescence intensity across biological triplicates. Note: Flow cytometry data were gated using the untreated (0 h) sample of each strain as the reference. The dotted vertical line indicates the mean fluorescence intensity (MFI) of the untreated control, representing the

baseline for GFP-negative cells. Shifts to the right of this line indicate increased MuGam-GFP or RecA-GFP signal relative to baseline. All samples were acquired using identical instrument settings, allowing valid comparison of fluorescence induction between strains. Data are shown as mean ± S.D. Gating was based on untreated (0 h) controls. At least 50,000 events were collected per sample.

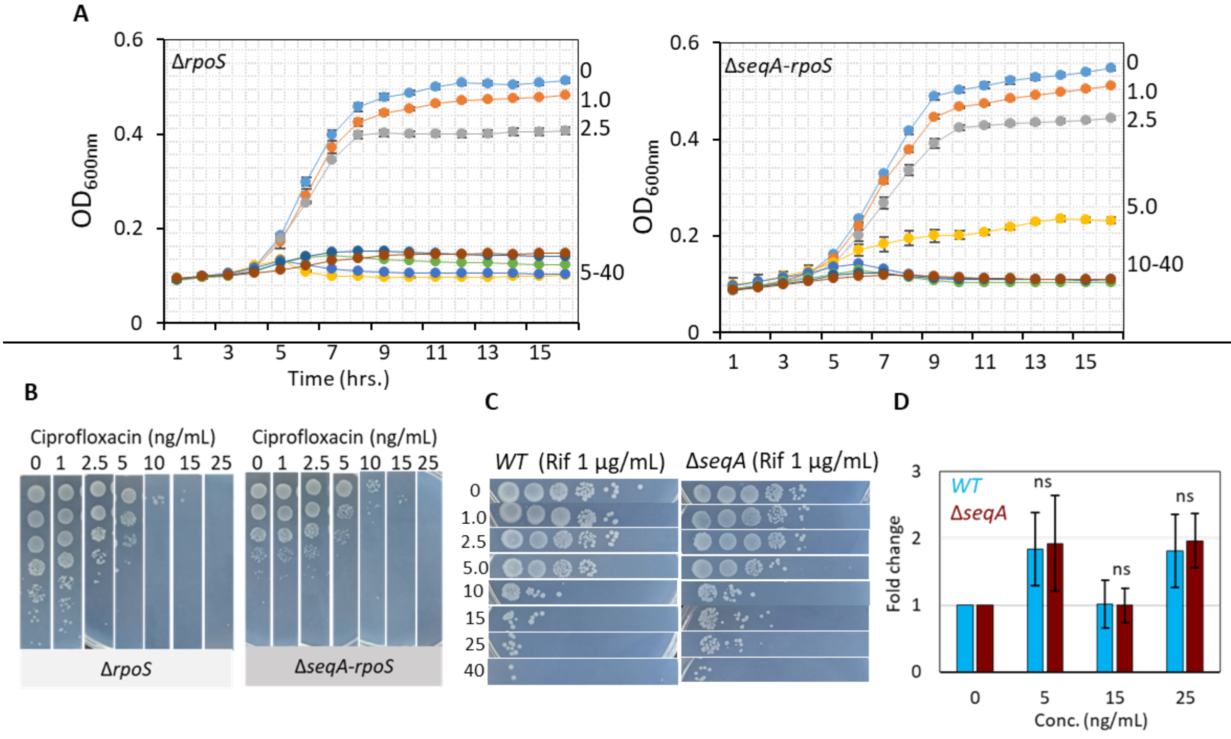

Figyre S7. Ciprofloxacin sensitivity of ΔrpoS and ΔseqAΔrpoS mutants. (A) Growth curves of *E. coli* Δ*rpoS* and Δ*seqA-rpoS* strains treated with increasing concentrations of ciprofloxacin (1, 2.5, 5, 10, 15, 25, and 40 ng/mL). (B) Spot dilution assay showing ciprofloxacin sensitivity of Δ*rpoS* and Δ*seqA-rpoS* strains (C) Spot dilution assay of *WT* and Δ*seqA* strains in the presence of ciprofloxacin gradient (0–40 ng/mL) with sub-MIC rifampicin (1 µg/mL). (D) Quantitative PCR analysis of *rpoS* gene-expression in *wildtype* and Δ*seqA* strains following 2-hour ciprofloxacin treatment; data normalized to *lacI*-gene expression and shown as fold change relative to untreated *wildtype.* Data represent mean ± SD of at least three independent biological replicates. Statistical significance was determined by Student's t-test (***p < 0.001).

Table S1. List of *E. coli* strains used in this study.

No.	Strain	Genotype/construction method	Source/Derivation
**1**	M35	MG1655	DB1607
**2**	MJ379	*ΔseqA::*tet^R	DB16
**3**	MJ460	*ΔrpoS*::Kan^R	Dipak Dutta IMtech
**4**	MJ1053	P1 MJ460 transduction in to MJ379	For this study
**5**	MJ609	PR-Gam-GFP, Cm^R	DB2467
**6**	MJ1264	P1 MJ609 transduction in to MJ379	For this study
**7**	MJ1051	RecA-GFP::Kan^R	Ox450
**8**	MJ1052	P1 MJ1051 transduction in to MJ379	For this study
**9**	MJ1130	GJ18609 PsulA :: (FRT-lacZY-kan-oriR6K-FRT)	J Gowrishankar CDFD
**10**	MJ1131	P1 MJ1130 transduction in to MJ379	For this study

Table S2. List of primers used for RT-qPCR.

Sr. No.	Primer	Forward primer	Reverse primer
1.	*rpoS gene*	GATGACGTCAGCCGTATGCTT	GAGGCCAATTTCACGACCTAC
2.	*lacI gene*	GGCGCTACA ACGGGTAGCAA	CAGCTTCCACAGCAATGGCAT
3.	*ydiJ gene*	CCACAGATTTCCCAGGC	AAGACGGGCGATCAG
4.	*gcvR gene*	GACACTGTCATCGCAAC	CGGCACCTTTCAACG

AIMS Press

© 2025 the Author(s), licensee AIMS Press. This is an open access article distributed under the terms of the Creative Commons Attribution License (http://creativecommons.org/licenses/by/4.0)
